# Supplementary material for: Proteomic Analysis of Synovial Fibroblasts and Articular Chondrocytes Co-Cultures Reveals Valuable VIP-Modulated Inflammatory and Degradative Proteins in Osteoarthritis
Source: Int J Mol Sci. 2021 Jun 16;22(12):6441. doi: 10.3390/ijms22126441 (PMC8235106; doi:10.3390/ijms22126441)
Supplement: Supplementary file 1 [file ijms-22-06441-s001.zip › ijms-1245412-supplementary.pdf]

Supplementary materials

Proteomic Analysis of Synovial Fibroblasts and Articular Chondrocytes Co-Cultures Reveals Valuable VIP-Modulated Inflammatory and Degradative Proteins in Osteoarthritis

Selene Pérez-García [1], Valentina Calamia [2], Tamara Hermida-Gómez [2], Irene Gutiérrez-Cañas [1], Mar Carrión [1], Raúl Villanueva-Romero [1], David Castro [1], Carmen Martínez [1], Yasmina Juarranz [1], Francisco J. Blanco [2] and Rosa P. Gomariz [1,*]

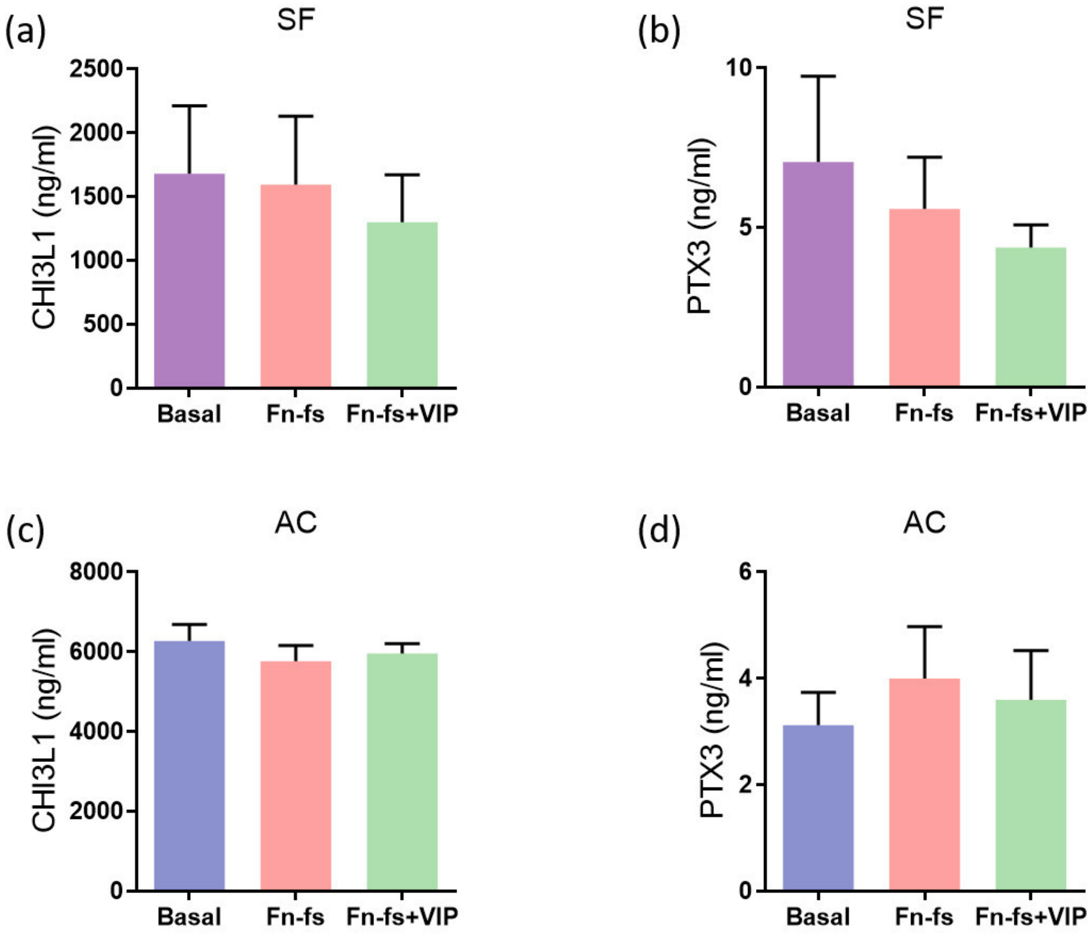

Figure S1. VIP-modulated inflammatory proteins. Protein secretion of (**a**),(**c**) CHI3L1 and (**b**),(**d**) PTX3 were determined by ELISA and Multiplex, respectively, in (**a**),(**b**) SF and (**c**),(**d**) AC cultures secretomes at 48 hours of treatment with and without 10 nM 45 kDa Fn-fs in the presence and absence of 10 nM VIP (n = 5). Data are presented as mean ± SEM of duplicate determinations.

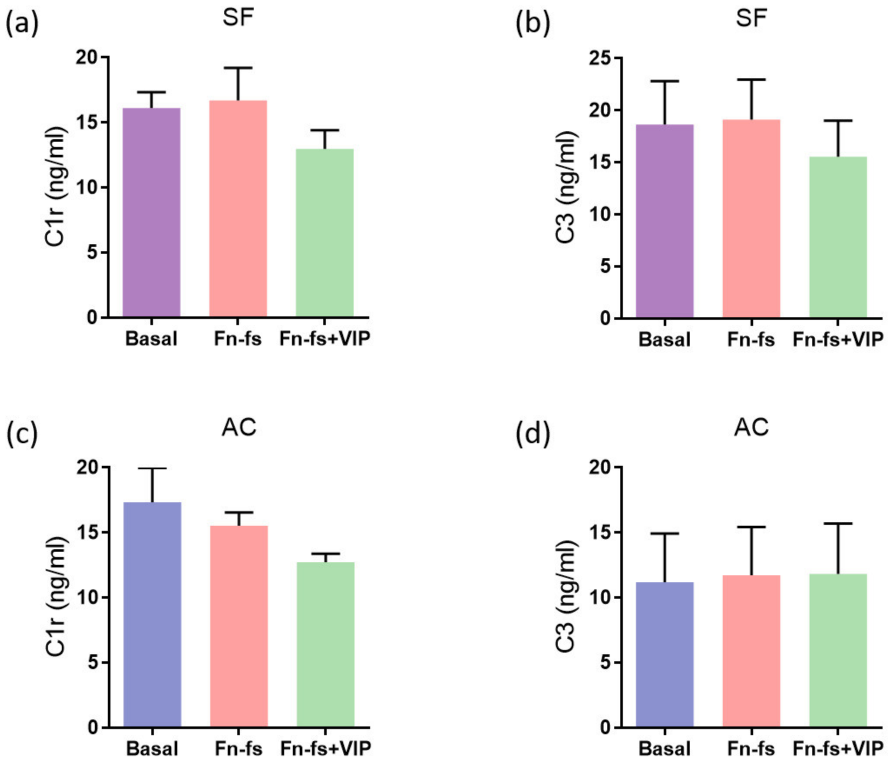

Figure S2. VIP-modulated complement pathway proteins. Protein secretion of (**a**),(**c**) C1R and (**b**),(**d**) C3 were determined by ELISA and Multiplex, respectively, in (**a**),(**b**) SF and (**c**),(**d**) AC cultures secretomes at 48 hours of treatment with and without 10 nM 45 kDa Fn-fs in the presence and absence of 10 nM VIP (n = 5). Data are presented as mean ± SEM of duplicate determinations.

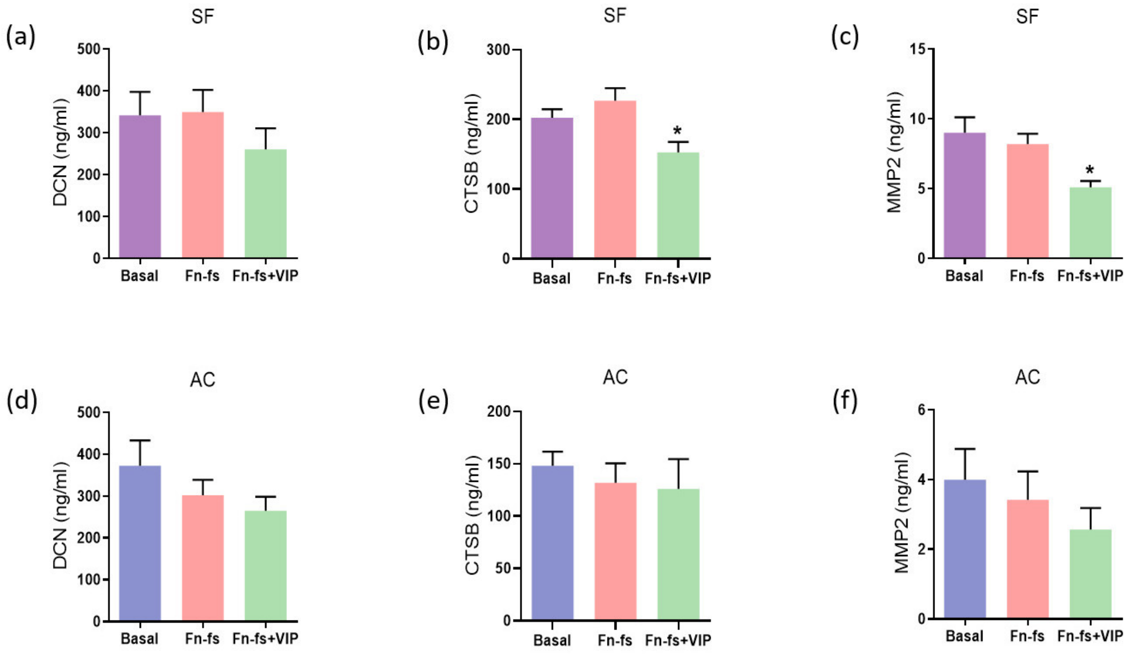

Figure S3. VIP-modulated ECM degradation proteins. Protein secretion of (**a**),(**d**) DCN, (**b**),(**e**) CTSB, and (**c**),(**f**) MMP2 were determined by ELISA and Multiplex, respectively, in (**a**),(**b**),(**c**) SF and (**d**),(**e**),(**f**) AC cultures secretomes at 48 hours of treatment with and without 10 nM 45 kDa Fn-fs in the presence and absence of 10 nM VIP (n = 5). Data are presented as mean ± SEM of duplicate determinations. *$p < 0.05$ Fn-fs+VIP vs Fn-fs.

Table S1. Proteins identified by SILAC analysis in the Fn-fs-stimulated secretome of OA SF-AC co-cultures with or without VIP treatment.

Forward

Acc No[a]	Protein Symbol	Protein Name	Peptides (95%)[b]	Ratio[c]	PVal[d]	EF[e]
P63261	ACTG	Actin, cytoplasmic 2	10	1.217	0.472	1.7964
P12814	ACTN1	Alpha-actinin-1	3	1.992	0.049	EF > 2
P04075	ALDOA	Fructe-bisphphate aldolase A	2	1.111	0.850	EF > 2
P15144	AMPN	Aminopeptidase N	1	0.779	0.861	EF > 2
P01008	ANT3	Antithrombin-III	2			
P27695	APEX1	DNA-(apurinic or apyrimidinic site) lyase	1	1.995		
Q15582	BGH3	Transforming growth factor-beta-induced protein ig-h3	5	0.686	0.109	1.6458
P00736	C1R	Complement C1r subcomponent	7	0.666	0.072	1.5743
P09871	C1S	Complement C1s subcomponent	5	0.939	0.824	1.8865
P40121	CAPG	Macrophage-capping protein	3	0.684	0.411	EF > 2
P07858	CATB	Cathepsin B	7	0.660	0.041	1.5426
P07339	CATD	Cathepsin D	1	0.950	0.990	EF > 2
P07711	CATL1	Cathepsin L1	3	0.698	0.220	EF > 2
P16070	CD44	CD44 antigen	1	1.174		
P00751	CFAB	Complement factor B	7	0.695	0.067	1.486
P08603	CFAH	Complement factor H	1	0.933		
P36222	CH3L1	Chitinase-3-like protein 1	46	0.481	0.000	1.32
P10909	CLUS	Clusterin	3	0.507	0.015	1.528
Q8N137	CNTRB	Centrobin	1			
P08123	CO1A2	Collagen alpha-2(I) chain	10	0.585	0.012	1.4872
P01024	CO3	Complement C3	1	0.545	0.022	1.4779
P12109	CO6A1	Collagen alpha-1(VI) chain	4	0.654	0.007	1.2983
P49747	COMP	Cartilage oligomeric matrix protein	4	0.665	0.041	1.4717
Q14019	COTL1	Coactin-like protein	2	1.558	0.141	EF > 2
O94985	CSTN1	Calsyntenin-1	1	0.709		
P13639	EF2	Elongation factor 2	2	1.745	0.187	EF > 2
P06733	ENOA	Alpha-enolase	7	1.640	0.001	1.2429
Q12805	FBLN3	EGF-containing fibulin-like extracellular matrix protein 1	1	0.414		
P02751	FINC	Fibronectin	11	0.552	0.552	EF > 2
Q06828	FMOD	Fibromodulin	2	0.595	0.147	EF > 2
P06744	G6PI	Gluce-6-phphate isomerase	1	1.833		
P07093	GDN	Glia-derived nexin	5	0.804	0.218	1.4731
P28161	GSTM2	Glutathione S-transferase Mu 2	1			
P09211	GSTP1	Glutathione S-transferase P	3	1.248	0.678	EF > 2
P02042	HBD	Hemoglobin subunit delta	1	0.010		
P11142	HSP7C	Heat shock cognate 71 kDa protein	1	2.408	0.076	EF > 2

P04792	HSPB1	Heat shock protein beta-1	2	1.586	0.505	EF > 2
P17936	IBP3	Insulin-like growth factor-binding protein 3	1	0.489	0.437	EF > 2
P22692	IBP4	Insulin-like growth factor-binding protein 4	2			
P24592	IBP6	Insulin-like growth factor-binding protein 6	1	0.684	0.185	EF > 2
Q16270	IBP7	Insulin-like growth factor-binding protein 7	2	1.291	0.235	EF > 2
P05155	IC1	Plasma protease C1 inhibitor	1	0.710		
P0DOX7	IGK	Immunoglobulin kappa light chain	1			
P05231	IL6	Interleukin-6	3	1.221	0.423	1.987 5
P13645	K1C10	Keratin, type I cytoskeletal 10	3			
P35527	K1C9	Keratin, type I cytoskeletal 9	2			
P04264	K2C1	Keratin, type II cytoskeletal 1	11	76.42 7		
P14618	KPYM	Pyruvate kinase PKM	5	1.389	0.158	1.646 5
P00338	LDHA	L-lactate dehydrogenase A chain	2	2.175	0.351	EF > 2
P17931	LEG3	Galectin-3	1	1.097	0.981	EF > 2
P51884	LUM	Lumican	10	0.536	0.008	1.511 3
P40925	MDHC	Malate dehydrogenase, cytoplasmic	1	3.158		
P03956	MMP1	Interstitial collagenase	17	0.967	0.888	1.671 8
P08253	MMP2	72 kDa type IV collagenase	10	0.672	0.031	1.419
P08254	MMP3	Stromelysin-1	7	1.386	0.725	EF > 2
P26038	MOES	Moesin	5	1.419	0.695	EF > 2
Q96TA1	NIBL1	Niban-like protein 1	1	1.167		
P05121	PAI1	Plasminogen activator inhibitor 1	1	0.620	0.143	EF > 2
Q15113	PCOC1	Procollagen C-endopeptidase enhancer 1	1	0.556		
P30101	PDIA3	Protein disulfide-isomerase A3	2	1.243	0.489	EF > 2
P18669	PGAM1	Phosphoglycerate mutase 1	2	1.324	0.340	EF > 2
P00558	PGK1	Phosphoglycerate kinase 1	1	1.083	0.834	EF > 2
P07585	PGS2	Decorin	14	0.582	0.008	1.454 3
P62937	PPIA	Peptidyl-prolyl cis-trans isomerase A	2	2.017	0.090	EF > 2
P23284	PPIB	Peptidyl-prolyl cis-trans isomerase B	1	1.653	0.377	EF > 2
Q06830	PRDX1	Peroxiredoxin-1	2	1.948	0.020	1.505 8
P30041	PRDX6	Peroxiredoxin-6	1	76.42 7		
P07737	PROF1	Profilin-1	1	1.033	0.969	EF > 2
P49721	PSB2	Proteasome subunit beta type-2	1	2.914		
Q9UL46	PSME2	Proteasome activator complex subunit 2	2	1.799	0.196	EF > 2
P26022	PTX3	Pentraxin-related protein 3	5	0.451	0.021	1.827 5
O00391	QSOX1	Sulfhydryl oxidase 1	6	0.610	0.003	1.302 3
P55017	S12A3	Solute carrier family 12 member 3	1			
P50454	SERPH	Serpin H1	1	1.368	0.379	EF > 2
P04179	SODM	Superoxide dismutase [Mn], mitochondrial	1	2.812		
P09486	SPRC	SPARC	3	0.526	0.031	1.644 1
P42224	STAT1	Signal transducer and activator of transcription 1-alpha/beta	2			
P23381	SYWC	Tryptophan--tRNA ligase, cytoplasmic	1	0.667	0.153	EF > 2

P68363	TBA1B	Tubulin alpha-1B chain	7	1.907	0.005	EF > 2
P07437	TBB5	Tubulin beta chain	5	2.630	0.012	1.909 8
P01033	TIMP1	Metalloproteinase inhibitor 1	1	0.566	0.270	EF > 2
P16035	TIMP2	Metalloproteinase inhibitor 2	1	0.608		
P29401	TKT	Transketolase	4	1.511	0.170	EF > 2
P60174	TPIS	Triephphate isomerase	2	0.806	0.530	EF > 2
P02788	TRFL	Lactotransferrin	1			
P19971	TYPH	Thymidine phphorylase	1	0.909		
P22314	UBA1	Ubiquitin-like modifier-activating enzyme 1	1	1.076	0.601	EF > 2
Q6EMK 4	VASN	Vasorin	1	0.429	0.077	EF > 2
P08670	VIME	Vimentin	16	1.758	0.402	EF > 2
P02774	VTDB	Vitamin D-binding protein	1			
P04004	VTNC	Vitronectin	1			
Q5GH72	XKR7	XK-related protein 7	1			

Reverse

Acc No[a]	Protein Symbo l	Protein Name	Peptide s (95%)[b]	Ratio [c]	PVal [d]	EF[e]
P01023	A2MG	Alpha-2-macroglobulin	2			
P63261	ACTG	Actin, cytoplasmic 2	8	1.022	0.780	1.194
P12814	ACTN 1	Alpha-actinin-1	4	1.462	0.143	1.842 4
P02768	ALBU	Serum albumin	25			
P04075	ALDO A	Fructe-bisphphate aldolase A	1	1.250		
P15144	AMPN	Aminopeptidase N	1	0.589		
P27695	APEX1	DNA-(apurinic or apyrimidinic site) lyase	1	1.084		
Q9P1U1	ARP3B	Actin-related protein 3B	1	1.202		
P61769	B2MG	Beta-2-microglobulin	1	0.823		
Q15582	BGH3	Transforming growth factor-beta-induced protein ig-h3	7	0.410	0.000	1.329 5
P00736	C1R	Complement C1r subcomponent	8	0.649	0.007	1.331 7
P09871	C1S	Complement C1s subcomponent	9	0.531	0.040	1.815 4
P40121	CAPG	Macrophage-capping protein	2	1.816	0.120	EF > 2
P07858	CATB	Cathepsin B	8	0.516	0.000	1.171 2
P07339	CATD	Cathepsin D	1	0.615	0.183	EF > 2
P07711	CATL1	Cathepsin L1	4	0.692	0.079	1.606 7
P16070	CD44	CD44 antigen	1	0.604		
P00751	CFAB	Complement factor B	10	0.591	0.191	EF > 2
P08603	CFAH	Complement factor H	1	1.419		
P36222	CH3L1	Chitinase-3-like protein 1	60	0.559	0.002	1.419 7

P10909	CLUS	Clusterin	5	0.880	0.907	EF > 2
P08123	CO1A2	Collagen alpha-2(I) chain	10	1.000	1.000	1.6512
P01024	CO3	Complement C3	1	0.346	0.064	EF > 2
P12109	CO6A1	Collagen alpha-1(VI) chain	3	0.886	0.465	1.4296
P12111	CO6A3	Collagen alpha-3(VI) chain	2	0.593	0.016	1.471
Q99715	COCA1	Collagen alpha-1(XII) chain	1	0.623		
P49747	COMP	Cartilage oligomeric matrix protein	9	1.222	0.508	1.6622
Q14019	COTL1	Coactin-like protein	2	1.314	0.253	EF > 2
P02511	CRYAB	Alpha-crystallin B chain	1	3.606		
O94985	CSTN1	Calsyntenin-1	1	0.839		
P13639	EF2	Elongation factor 2	1	1.514	0.181	EF > 2
P06733	ENOA	Alpha-enolase	5	1.133	0.589	1.7091
Q12805	FBLN3	EGF-containing fibulin-like extracellular matrix protein 1	1	0.373		
P02751	FINC	Fibronectin	12	0.568	0.013	1.5137
Q06828	FMOD	Fibromodulin	2	0.818	0.631	EF > 2
P04406	G3P	Glyceraldehyde-3-phphate dehydrogenase	1	1.292	0.235	EF > 2
P06744	G6PI	Gluce-6-phphate isomerase	1	1.369		
P50395	GDIB	Rab GDP dissociation inhibitor beta	1	1.459	0.829	EF > 2
P07093	GDN	Glia-derived nexin	9	0.431	0.005	1.6508
P09211	GSTP1	Glutathione S-transferase P	2	1.880	0.099	EF > 2
P57053	H2BFS	Histone H2B type F-S	1	2.068		
P02042	HBD	Hemoglobin subunit delta	1			
P04792	HSPB1	Heat shock protein beta-1	1	1.729	0.221	EF > 2
P17936	IBP3	Insulin-like growth factor-binding protein 3	2	1.003	0.995	EF > 2
P22692	IBP4	Insulin-like growth factor-binding protein 4	2	1.057	0.704	EF > 2
P24592	IBP6	Insulin-like growth factor-binding protein 6	1	0.576		
Q16270	IBP7	Insulin-like growth factor-binding protein 7	2	0.787	0.249	EF > 2
P0DOX7	IGK	Immunoglobulin kappa light chain	1			
P05231	IL6	Interleukin-6	4	0.424	0.032	EF > 2
P13645	K1C10	Keratin, type I cytoskeletal 10	2			
P35527	K1C9	Keratin, type I cytoskeletal 9	2			
P35908	K22E	Keratin, type II cytoskeletal 2 epidermal	3	0.010		
P04264	K2C1	Keratin, type II cytoskeletal 1	10	0.010		

P14618	KPYM	Pyruvate kinase PKM	4	1.204	0.522	1.996 6
P00338	LDHA	L-lactate dehydrogenase A chain	3	1.515	0.466	EF > 2
P51884	LUM	Lumican	16	0.632	0.253	EF > 2
P33908	MA1A 1	Mannyl-oligaccharide 1,2-alpha-mannidase IA	1	0.589		
P14174	MIF	Macrophage migration inhibitory factor	2	0.775		
P03956	MMP1	Interstitial collagenase	18	0.322	0.014	EF > 2
P08253	MMP2	72 kDa type IV collagenase	10	0.541	0.003	1.400 1
P08254	MMP3	Stromelysin-1	10	0.414	0.082	EF > 2
P26038	MOES	Moesin	5	1.344	0.206	1.686 5
P22392	NDKB	Nucleide diphphate kinase B	2	1.949	0.135	EF > 2
Q96TA1	NIBL1	Niban-like protein 1	1	2.583		
P05121	PAI1	Plasminogen activator inhibitor 1	1	0.542	0.105	EF > 2
Q15113	PCOC1	Procollagen C-endopeptidase enhancer 1	1	0.366		
P30101	PDIA3	Protein disulfide-isomerase A3	4	2.090	0.003	1.352 6
Q15084	PDIA6	Protein disulfide-isomerase A6	1			
P18669	PGAM 1	Phosphoglycerate mutase 1	2	1.994	0.112	EF > 2
P21810	PGS1	Biglycan	1	0.554	0.106	EF > 2
P07585	PGS2	Decorin	13	0.540	0.022	1.665
O60664	PLIN3	Perilipin-3	1	2.891		
P62937	PPIA	Peptidyl-prolyl cis-trans isomerase A	2	1.573	0.145	EF > 2
P23284	PPIB	Peptidyl-prolyl cis-trans isomerase B	1	1.110	0.852	EF > 2
Q06830	PRDX1	Peroxiredoxin-1	1	1.725	0.393	EF > 2
P30041	PRDX6	Peroxiredoxin-6	1	1.014		
Q92954	PRG4	Proteoglycan 4	2	0.831	0.737	EF > 2
P49721	PSB2	Proteasome subunit beta type-2	1	1.289		
Q9UL46	PSME2	Proteasome activator complex subunit 2	2	2.023	0.296	EF > 2
P26022	PTX3	Pentraxin-related protein 3	5	0.628	0.069	1.783 1
O00391	QSOX1	Sulfhydryl oxidase 1	6	0.474	0.001	1.415 6
P50454	SERPH	Serpin H1	2	1.497	0.161	EF > 2
Q9H299	SH3L3	SH3 domain-binding glutamic acid-rich-like protein 3	1	2.518		
P04179	SODM	Superoxide dismutase [Mn], mitochondrial	2	0.853		
P09486	SPRC	SPARC	5	0.551	0.239	EF > 2
P23381	SYWC	Tryptophan--tRNA ligase, cytoplasmic	1	0.914		

P68363	TBA1B	Tubulin alpha-1B chain	8	1.511	0.017	1.379 9
Q13509	TBB3	Tubulin beta-3 chain	4	1.841	0.532	EF > 2
P07437	TBB5	Tubulin beta chain	4	1.598	0.717	EF > 2
P24821	TENA	Tenascin	1	0.635	0.274	EF > 2
P01033	TIMP1	Metalloproteinase inhibitor 1	4	0.654	0.023	1.403
P16035	TIMP2	Metalloproteinase inhibitor 2	1	0.753	0.214	EF > 2
P29401	TKT	Transketolase	3	1.398	0.050	1.553 7
P60174	TPIS	Triephphate isomerase	3	1.600	0.195	EF > 2
P02787	TRFE	Serotransferrin	2	0.010		
Q8NBS 9	TXND 5	Thioredoxin domain-containing protein 5	2	1.343	0.210	EF > 2
P19971	TYPH	Thymidine phphorylase	1	1.039		
P22314	UBA1	Ubiquitin-like modifier-activating enzyme 1	1	2.945		
O60701	UGDH	UDP-glucose 6-dehydrogenase	1	5.006	0.145	EF > 2
Q6EMK 4	VASN	Vasorin	1	0.717	0.335	EF > 2
P08670	VIME	Vimentin	11	1.230	0.275	1.48
P18206	VINC	Vinculin	2	1.776		
P04004	VTNC	Vitronectin	1			

[a]Protein accession number according to the SwissProt and TrEMBL databases. [b]Number of unique peptides (Pept) used for protein identification at 95% confidence. [c]SILAC ratios that represent the relative protein abundance in Fn-fs+VIP versus Fn-fs treated SF-AC co-cultures at the same time point (48 h) (n = 4). [d]*p*-value of the corresponding SILAC ratios. [e]Error factor calculated by Protein Pilot 4.0 software for quantification accuracy. SILAC, stable isotope labelling by amino acids in cell culture; OA SF-AC, osteoarthritic synovial fibroblasts-articular chondrocytes.

Table S2. Proteins identified by SILAC analysis in the secretome of OA Fn-fs-stimulated SF-AC co-cultures and normal IL-1β-stimulated AC.

Acc No[a]	Protein Symbol	Protein name	Fn-fs stimulated SF-AC[b]	IL-1β stimulated AC[c]
P01009	A1AT	Alpha-1-antitrypsin		x
P01023	A2MG	Alpha-2-macroglobulin	x	
P63261	ACTG	Actin, cytoplasmic 2	x	
P12814	ACTN1	Alpha-actinin-1	x	
P04075	ALDOA	Fructe-bisphphate aldolase A	x	
P15144	AMPN	Aminopeptidase N	x	
P01008	ANT3	Antithrombin-III	x	
P07355	ANXA2	Annexin A2		x
P27695	APEX1	DNA-(apurinic or apyrimidinic site) lyase	x	
Q9P1U 1	ARP3B	Actin-related protein 3B	x	
P61769	B2MG	Beta-2-microglobulin	x	x
Q15582	BGH3	Transforming growth factor-beta-induced protein ig-h3	x	x
P00736	C1R	Complement C1r subcomponent	x	x
P09871	C1S	Complement C1s subcomponent	x	x
P40121	CAPG	Macrophage-capping protein	x	
P07858	CATB	Cathepsin B	x	x
P07339	CATD	Cathepsin D	x	
P07711	CATL1	Cathepsin L1	x	
Q76M9 6	CCD80	Coiled-coil domain-containing protein 80		x
P13500	CCL2	C-C motif chemokine 2b		x
P80075	CCL8	C-C motif chemokine 8b		x
P16070	CD44	CD44 antigen	x	
P00751	CFAB	Complement factor B	x	x
P08603	CFAH	Complement factor H	x	
P36222	CH3L1	Chitinase-3-like protein 1	x	x
Q15782	CH3L2	Chitinase-3-like protein 2b		x
P10909	CLUS	Clusterin	x	x
Q8N137	CNTRB	Centrobin	x	
P08123	CO1A2	Collagen alpha-2(I) chain	x	x
P01024	CO3	Complement C3	x	x
P02461	CO3A1	Collagen alpha-1(III) chain		x
P12109	CO6A1	Collagen alpha-1(VI) chain	x	x
P12111	CO6A3	Collagen alpha-3(VI) chain	x	
Q99715	COCA1	Collagen alpha-1(XII) chain	x	x
P49747	COMP	Cartilage oligomeric matrix protein	x	
Q14019	COTL1	Coactin-like protein	x	
P02511	CRYAB	Alpha-crystallin B chain	x	
P09603	CSF1	Macrophage colony-stimulating factor 1b		x
O94985	CSTN1	Calsyntenin-1	x	
P19876	CXCL3	C-X-C motif chemokine 3b		x
P42830	CXCL5	C-X-C motif chemokine 5b		x
P80162	CXCL6	C-X-C motif chemokine 6b		x
P01034	CYTC	Cystatin-C		x

P15924	DESP	Desmoplakinb		x
P13639	EF2	Elongation factor 2	x	
P06733	ENOA	Alpha-enolase	x	
P23142	FBLN1	Fibulin-1		x
Q12805	FBLN3	EGF-containing fibulin-like extracellular matrix protein 1	x	x
P02751	FINC	Fibronectin	x	x
Q06828	FMOD	Fibromodulin	x	
Q12841	FSTL1	Follistatin-related protein 1		x
P04406	G3P	Glyceraldehyde-3-phphate dehydrogenase	x	
P06744	G6PI	Gluce-6-phphate isomerase	x	
P50395	GDIB	Rab GDP dissociation inhibitor beta	x	
P07093	GDN	Glia-derived nexin	x	x
P09341	GROA	Growth-regulated alpha protein		x
P28161	GSTM2	Glutathione S-transferase Mu 2	x	
P09211	GSTP1	Glutathione S-transferase P	x	
P57053	H2BFS	Histone H2B type F-S	x	
P02042	HBD	Hemoglobin subunit delta	x	
P11142	HSP7C	Heat shock cognate 71 kDa protein	x	
P04792	HSPB1	Heat shock protein beta-1	x	
P17936	IBP3	Insulin-like growth factor-binding protein 3	x	x
P22692	IBP4	Insulin-like growth factor-binding protein 4	x	x
P24593	IBP5	Insulin-like growth factor-binding protein 5		x
P24592	IBP6	Insulin-like growth factor-binding protein 6	x	x
Q16270	IBP7	Insulin-like growth factor-binding protein 7	x	x
P05155	IC1	Plasma protease C1 inhibitor	x	x
P0DOX 7	IGK	Immunoglobulin kappa light chain	x	
P05231	IL6	Interleukin-6	x	x
P10145	IL8	Interleukin-8		x
P13645	K1C10	Keratin, type I cytoskeletal 10	x	x
P02533	K1C14	Keratin, type I cytoskeletal 14b		x
P08779	K1C16	Keratin, type I cytoskeletal 16b		x
P35527	K1C9	Keratin, type I cytoskeletal 9	x	x
P04264	K2C1	Keratin, type II cytoskeletal 1	x	x
P04259	K2C6B	Keratin, type II cytoskeletal 6Bb		x
P14618	KPYM	Pyruvate kinase PKM	x	
P00338	LDHA	L-lactate dehydrogenase A chain	x	
P17931	LEG3	Galectin-3	x	
P51884	LUM	Lumican	x	x
P33908	MA1A1	Mannyl-oligaccharide 1,2-alpha-mannidase IA	x	
P40925	MDHC	Malate dehydrogenase, cytoplasmic	x	
Q08431	MFGM	Lactadherin		x
P14174	MIF	Macrophage migration inhibitory factor	x	
P03956	MMP1	Interstitial collagenase	x	x
P08253	MMP2	72 kDa type IV collagenase	x	x
P08254	MMP3	Stromelysin-1	x	x
P26038	MOES	Moesin	x	

P22392	NDKB	Nucleide diphphate kinase B	x	
Q96TA 1	NIBL1	Niban-like protein 1	x	
Q8N130	NPT2C	Sodium-dependent phosphate transport protein 2Cb		x
P05121	PAI1	Plasminogen activator inhibitor 1	x	
Q15113	PCOC1	Procollagen C-endopeptidase enhancer 1	x	x
P30101	PDIA3	Protein disulfide-isomerase A3	x	
Q15084	PDIA6	Protein disulfide-isomerase A6	x	
P18669	PGAM1	Phosphoglycerate mutase 1	x	
P00558	PGK1	Phosphoglycerate kinase 1	x	
P21810	PGS1	Biglycan	x	x
P07585	PGS2	Decorin	x	x
O60664	PLIN3	Perilipin-3	x	
P55058	PLTP	Phospholipid transfer protein		x
A5A3E 0	POTEF	POTE ankyrin domain family member Fb		x
P62937	PPIA	Peptidyl-prolyl cis-trans isomerase A	x	
P23284	PPIB	Peptidyl-prolyl cis-trans isomerase B	x	
Q06830	PRDX1	Peroxiredoxin-1	x	
P30041	PRDX6	Peroxiredoxin-6	x	
Q92954	PRG4	Proteoglycan 4	x	x
P07737	PROF1	Profilin-1	x	
P49721	PSB2	Proteasome subunit beta type-2	x	
Q9UL4 6	PSME2	Proteasome activator complex subunit 2	x	
P26022	PTX3	Pentraxin-related protein 3	x	x
O00391	QSOX1	Sulfhydryl oxidase 1	x	x
P05109	S10A8	Protein S100-A8b		x
P06702	S10A9	Protein S100-A9b		x
P55017	S12A3	Solute carrier family 12 member 3	x	
P07602	SAP	Proactivator polypeptideb		x
P31431	SDC4	Syndecan-4		x
P50454	SERPH	Serpin H1	x	
Q9H299	SH3L3	SH3 domain-binding glutamic acid-rich-like protein 3	x	
P04179	SODM	Superoxide dismutase [Mn], mitochondrial	x	
P09486	SPRC	SPARC	x	x
P42224	STAT1	Signal transducer and activator of transcription 1-alpha/beta	x	
P23381	SYWC	Tryptophan--tRNA ligase, cytoplasmic	x	
Q7Z7G 0	TARSH	Target of Nesh-SH3		x
P68363	TBA1B	Tubulin alpha-1B chain	x	
Q13509	TBB3	Tubulin beta-3 chain	x	
P07437	TBB5	Tubulin beta chain	x	
P24821	TENA	Tenascin	x	x
P05452	TETN	Tetranectin		x
P01033	TIMP1	Metalloproteinase inhibitor 1	x	x
P16035	TIMP2	Metalloproteinase inhibitor 2	x	x
P29401	TKT	Transketolase	x	

P60174	TPIS	Triephphate isomerase	x	
P02787	TRFE	Serotransferrin	x	
P02788	TRFL	Lactotransferrin	x	
Q8NH M4	TRY6	Putative trypsin-6b		x
P98066	TSG6	TNF-inducible gene 6 protein		x
P07996	TSP1	Thrombospondin-1		x
Q8NBS 9	TXND5	Thioredoxin domain-containing protein 5	x	
P19971	TYPH	Thymidine phphorylase	x	
P22314	UBA1	Ubiquitin-like modifier-activating enzyme 1	x	
O60701	UGDH	UDP-gluce 6-dehydrogenase	x	
Q6EMK 4	VASN	Vasorin	x	x
P19320	VCAM1	Vascular cell adhesion protein 1		x
P08670	VIME	Vimentin	x	
P18206	VINC	Vinculin	x	
P02774	VTDB	Vitamin D-binding protein	x	
P04004	VTNC	Vitronectin	x	
Q5GH7 2	XKR7	XK-related protein 7	x	

[a]Protein accession number according to the SwissProt and TrEMBL databases. [b]Proteins identified by SILAC analysis in the present study. [c]Proteins identified by SILAC analysis in the previous study [32]. SILAC, stable isotope labelling by amino acids in cell culture; OA, osteoarthritis; Fn-fs, fibronectin fragments; SF, synovial fibroblasts; AC, articular chondrocytes; IL-1β, interleukin-1β.
